# Research on the Effect Mechanism of Re on Interface Dislocation Networks of Ni–Based Single Crystal Alloys

**DOI:** 10.3390/ma17102361

**Published:** 2024-05-15

**Authors:** Ben Li, Hongyan Zhou

**Affiliations:** 1 Engineering Research Center of Additive Manufacturing Aeronautical Materials of Henan Province, Nanyang Institute of Technology, Nanyang 473004, China; zhou_2510@163.com; 2 School of Mechanical and Power Engineering, East China University of Science and Technology, Shanghai 200237, China

**Keywords:** molecular dynamics, interface dislocation networks, lamb wave

## Abstract

The effect of interface dislocation networks on the mechanical properties of new Ni–based single crystal alloys containing Rhenium (Re) is very large. Because the interface dislocations are microscopic in the nano–scale range, this has not been investigated, and it is very difficult to prepare new Ni–based single crystal alloys containing Re. Therefore, six kinds of new Ni–based single crystal alloys containing Re were prepared, and the hardness tests and nonlinear ultrasonic lamb wave tests were performed on the samples. It was found that the density of interface dislocation networks increases with the increase in the content of Re, which improves the blocking ability of matrix phase dislocation cutting into precipitated phase and enhances the inhibition of dislocation movement. The nonlinear ultrasonic lamb wave tests showed that the materials exhibit better mechanical properties when the density of the interface dislocation networks increases. Meanwhile, a new molecular dynamics model which is closer to the real state of an Ni–based single crystal alloy was constructed to reveal the evolution mechanism of interface dislocation networks. The results showed that the potential energy of Re atoms at the interface is the lowest, which affects the reduction of the potential energy of other atoms at the interface, and thus the stability of the model is improved. In addition, according to the change in the total length of dislocation loops in the model system, with the increase in the content of Re atoms, the inhibition of dislocation movement by dislocation networks at the interface is strengthened.

## 1. Introduction

Re is a rare metal element with a melting point of 3180 °C and high temperature mechanical properties; thus, it can be used to prepare advanced aero–engine turbine blades. At present, the basic capabilities of the advanced fifth–generation fighter plane are super mobility and supersonic cruise. However, there are only two models currently capable of achieving these capabilities worldwide, the F22 fighter plane with an F119 engine and the J20 fighter plane with a WS15 engine, which both use Ni–based single crystal turbine blades containing Re [1].

Re can improve the mechanical properties (fatigue and creep life) and increase the working temperature of Ni–based single crystal turbine blades. The literature [1] showed that for every 50 °C increase in the turbine inlet temperature, the corresponding engine thrust could be increased by 10%. The latest version of F119 has a maximum turbine inlet temperature of 1677 °C, thrust of 180 kN and a lifetime of 8000 h after the integration of Re. This explains why the F–22 is the only fighter plane in the U.S. Air Force that can supercruise without using afterburners and why its air–to–air capabilities are so impressive. The thrust of the WS15 engine turbine blade is greatly increased after Re is integrated, and its service life is much longer than that of WS10A/B. Accordingly, an Ni–based single crystal turbine blade containing Re is the key factor to improve the performance of the engine. However, the production of Ni–based single crystal alloys containing Re is very difficult. Nowadays, only two countries in the world have the capacity to mass produce Ni–based single crystal alloys containing Re for turbine blades. This is mainly because of the lack of in–depth understanding of Ni–based single crystal alloys containing Re at the micro level (micro–defects affect the quality of preparation), especially the study of the effect mechanism of Re on the interface dislocation networks of Ni–based single crystal alloys at the nano scale.

At present, studies of Ni–based single crystal alloys containing Re are mostly focused on the macroscopic scale and the characterization of macroscopic mechanical properties. The creep resistance of Ni–based single crystal alloys containing Re at 1100 °C /137 MPA was analyzed in detail in the literature [2], and the results showed that with the increase in the content of Re, the hindrance to the dislocation entering the γ′ phase was stronger. In addition, Re atoms could replace Al atoms, which increased the bonding force between atoms, thus making the creep life of Ni–based single crystal alloys increase from 164 h to 321 h. Studies of Ni–based single crystal alloys at the micro level almost never contain Re. The related studies in the literature [3,4,5] used a DD6 Ni–based single crystal alloy as the sample material, which does not contain Re. 

Based on the above analysis, there is little research on the molecular dynamics of Ni–based single crystal alloys containing Re, especially the effect mechanism of Re on the interface dislocation networks of Ni–based single crystal alloys. Therefore, this study will use experimental tests and the molecular dynamics method to carry out a detailed investigation. 

## 2. Experimental Tests and Molecular Dynamics Simulation

In this study, the hardness of the samples was tested by a diamond indenter, and the molecular dynamics method was used for modeling the analysis, as discussed in detail in the following sections. OVITO 3.4.4 software was used to carry out the analysis, and the nonlinear ultrasonic lamb wave was used to assist the analysis. Accordingly, the microstructural evolution behavior of the interface dislocation networks of an Ni–based single crystal alloy was studied. 

## 3. Results and Discussion

**Figure 1:**
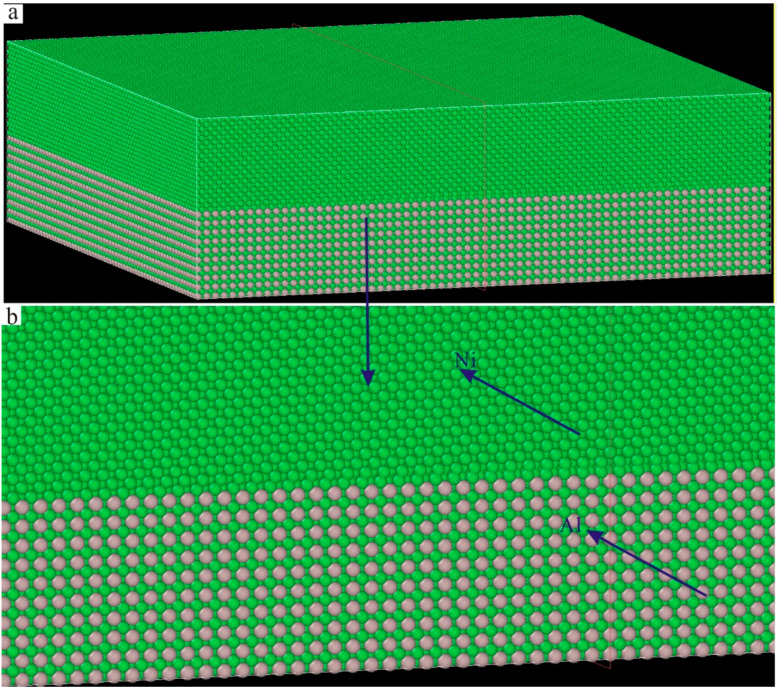
Traditional sandwich model of Ni–based single crystal alloys: (**a**) three–dimensional model; (**b**) local magnification of the three–dimensional model.

In order to study the effect of Re on the interface dislocation networks of Ni–based single crystal alloys, six kinds of Ni–based single crystal alloy samples with different contents of Re (0 wt.%, 1 wt.%, 2 wt.%, 3 wt.%, 4 wt.% and 5 wt.%) were prepared (Table 1), and then they were mechanically ground and polished for the following tests. The Vicker’s hardness of each sample was tested and analyzed using a NEMESIS 9100 general purpose hardness tester (Produced by INNOVATEST in the Netherlands), and the mechanical properties of each sample were investigated by means of nonlinear ultrasonic lamb wave tests. In addition, for the purpose of revealing the effect of Re on the interface dislocation networks of an Ni–based crystal alloy at the nano scale, the molecular dynamics method was used to study the microstructural evolution behavior of interface dislocation networks under compressive loading. At present, most molecular dynamics simulations of Ni–based single crystal alloys use an Ni–Al sandwich model (see Figure 1), as only Ni and Al can be found in the three–dimensional model. However, this Ni–Al sandwich model is quite different from the actual structure of the new Ni–based single crystal alloys containing Re.

The effect of Re on the mechanical properties of Ni–based single crystal alloys is large and can be greatly improved by adding a proper amount of Re. The Re atom decreases the atomic potential energy of the γ/γ′ interface dislocation network region, which can enhance the stability of the γ/γ′ interface dislocation network and the blocking ability of matrix phase (γ phase) dislocations cutting into the precipitated phase (γ′ phase), leading to the improved plastic yield resistance of Ni–based single crystal alloys. 

The molar mass of an Re atom (about 186.21 g/mol) is 3.17 times that of an Ni atom, and it has strong solid solution strengthening. The results in the literature [6] showed that when the volume ratio of γ phase to γ′ phase was between 60% and 70%, the dislocation movement of γ phase was greatly restricted by the integration of Re atoms, and the tensile strength and creep resistance were improved. Therefore, it is necessary to consider the volume ratio of the γ phase to the γ′ phase when constructing a new molecular dynamics model of an Ni–based single crystal alloy containing Re. There is no Re in the traditional sandwich model in Figure 1, and the model is too small (only 353,800 atoms) to show the true properties of the single crystal alloys. At the same time, the lattice constant of the γ phase $a_{\gamma }$ = 0.352 nm, and the lattice constant of the γ′ phase $a_{\gamma \prime }$ = 0.3567 nm in Ni–based single crystal alloys. Therefore, there must be mismatches at the interface between the two phases:

(1) $$\delta =\frac{2\left(a_{\gamma^{\prime }}-a_{\gamma }\right)}{\left(a_{\gamma^{\prime }}+a_{\gamma }\right)}\;\;$$

According to the results in the literature [7,8], the relationship between *γ* and *γ*′ is as follows:

(2) $$na_{\gamma \prime }=\left(n+1\right)a_{\gamma }$$

**Figure 2:**
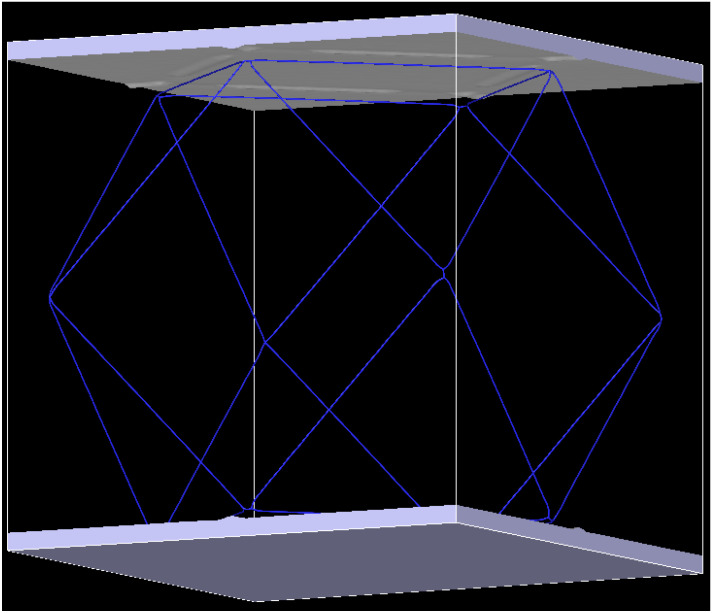
Interface dislocation network of Ni–based single crystal alloy.

From Equation (2), *n* = 75. Accordingly, a cube–type Ni–based single crystal alloy model can be established. The size of the model is 302.72 Å × 302.72 Å × 302.72 Å, the coordinate origin is in the center of the model and the corresponding interface dislocation network is shown in Figure 2. In this square interface dislocation network, there are 34 total dislocations in the relaxed model system, and the total length of dislocation loop is 4515.62 Å. 

**Figure 3:**
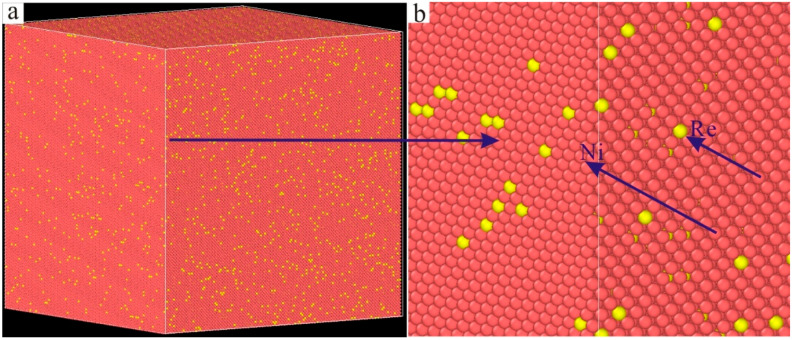
Molecular dynamics model of the new Ni–based single crystal alloy containing Re: (**a**) three–dimensional model; (**b**) local magnification of the three–dimensional model.

**Figure 4:**
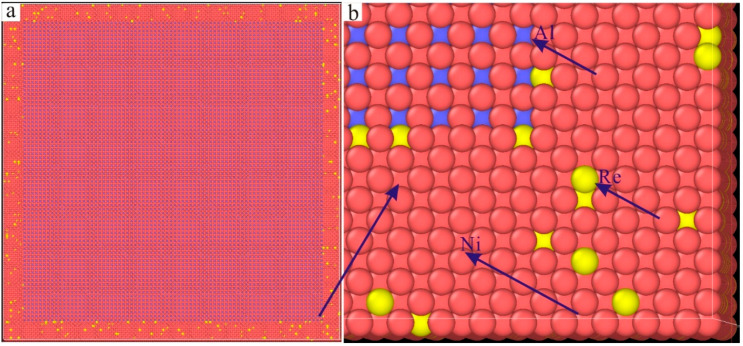
Slice analysis of molecular dynamics model of the new Ni–based single crystal alloy containing Re: (**a**) section view; (**b**) local magnification of the section view.

The molecular dynamics model of the new Ni–based single crystal alloy containing Re is shown in Figure 3; the yellow atoms are Re, the red atoms are Ni and the atoms of the γ phase are randomly replaced by Re atoms. This molecular dynamics model (001 crystal phase) has a clear cube configuration, and the Re atoms are randomly distributed in the model, which is very close to the Re atom distribution in the actual Ni–based single crystal alloys. However, the Al atoms cannot be observed in Figure 3. In order to show the distribution of Ni, Al and Re atoms, the three–dimensional model was analyzed by cutting into sections, as shown in Figure 4, where the blue atoms are Al atoms. To construct the molecular dynamics model of an Ni–based single crystal alloy containing Re, a new model (Figure 3) was used, in which the γ phase was outside of the model, while the γ′ phase is inside of the model, and the volume ratio of the γ phase to the γ′ phase was close to three to seven. Moreover, the number of atoms in the model exceeds 2 million (2,474,911 in Figure 3). In a word, the molecular dynamics model of the new Ni–based single crystal alloy containing Re is closer to the real properties of advanced Ni–based single crystal alloy materials. 

**Figure 5:**
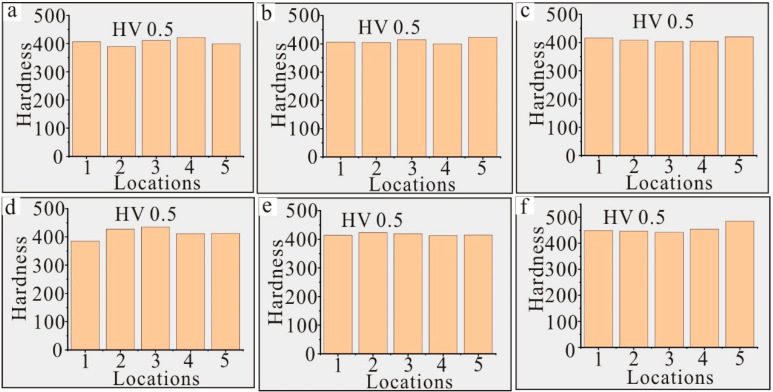
Hardness test results of Ni–based single crystal alloys containing Re: (**a**) 0 wt.% Re; (**b**) 1 wt.% Re; (**c**) 2 wt.% Re; (**d**) 3 wt.% Re; (**e**) 4 wt.% Re; (**f**) 5 wt.% Re.

The hardness test results of sample 1 containing 0 wt.% Re at HV (Vickers Hardness) 0.5 are shown in Figure 5a. The tests were carried out at five different locations to reduce random errors, and the average hardness was 405. The hardness test results of sample 2 containing 1 wt.% Re at HV 0.5 are shown in Figure 5b. The average hardness was 410, indicating that with the increase in the content of Re, the hardness of the Ni–based single crystal alloy increases, which is mainly because of the solid solution strengthening of Re [6]. The hardness test results of sample 3 containing 2 wt.% Re, sample 4 containing 3 wt.% Re and sample 5 containing 4 wt.% Re at HV 0.5 are shown in Figure 5c–e, respectively, and the corresponding average hardnesses were 411, 414 and 417, respectively. When the content of Re increased from 2 to 4 wt.%, the hardnesses of the samples showed an increasing trend, but the increase was not significant. The hardness test results of sample 6 containing 5 wt.% Re at HV 0.5 are shown in Figure 5f; the average value increased significantly from 417 to 455. According to the above analysis, the hardnesses of Ni–based single crystal alloys obviously increases with the increasing in Re (if the content of Re is less than 6 wt.%). 

**Figure 6:**
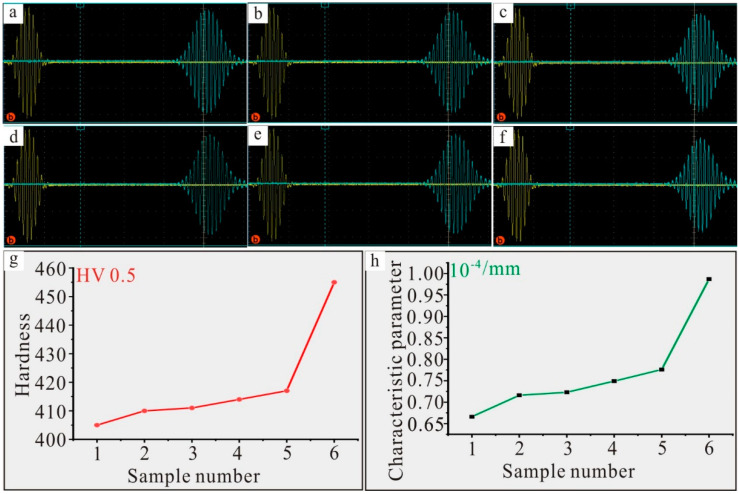
(**a**) Time–domain signal of sample 1; (**b**) Time–domain signal of sample 2; (**c**) Time–domain signal of sample 3; (**d**) Time–domain signal of sample 4; (**e**) Time–domain signal of sample 5; (**f**) Time–domain signal of sample 6; (**g**) Change trend of the hardnesses of prepared samples; (**h**) Change trend of the nonlinear characteristic parameters of prepared samples.

The theoretical relationship between the quality of Ni–based single crystal alloys and the microstructural evolution of the nonlinear ultrasonic lamb wave has been described in detail in the literature [9]. However, the qualitative expression relationship between hardness and the nonlinear characteristic parameter has not been found in the literature [9]; thus, the prepared samples were tested using a nonlinear ultrasonic lamb wave detection system (RAM–5000–SNAP). The optimum excitation frequency was 2.07 MHz (the angle of incidence and receiving was 19.6°) after the calculation and analysis of corresponding dispersion curves. After testing, the corresponding excitation and reception signals of the lamb wave are shown in Figure 6a–f (The picture is automatically generated, the left side is the transmitting signal, and the right side is the receiving signal. The signal cannot be directly interpreted on the picture, and signal analysis and processing are required). Figure 6a shows the time–domain signal of sample 1, and after signal analysis and processing, the corresponding nonlinear characteristic parameter was 0.666 × 10^−4^/mm. Figure 6b shows the corresponding time–domain signal of sample 2. With the increase in the content of Re, the nonlinear acoustic contact effect of Ni–based single crystal alloys increased; thus, the corresponding nonlinear characteristic parameter increased to 0.716 × 10^−4^/mm. Figure 6c–e shows the time–domain signals of sample 3, sample 4 and sample 5, respectively, and the corresponding nonlinear characteristic parameters were 0.723 × 10^−4^/mm, 0.749 × 10^−4^/mm and 0.776 × 10^−4^/mm, respectively. With the increase in the content of Re, the corresponding nonlinear characteristic parameter also increased. Figure 6f shows the time–domain signal of sample 6; the corresponding nonlinear characteristic parameter was greatly increased to 0.987 × 10^−4^/mm. This could be attributed to the fact that the increase in the content of Re improves the density of the interface dislocation network, and the nonlinear characteristic parameter is proportional to the dislocation density [3]. According to the above analysis, with the increase in the content of Re, the change trend in the hardnesses of the prepared samples was consistent with that of the nonlinear characteristic parameters (see Figure 6g,h). This finding will provide a technical reference for further research on the quality evaluation of new Ni–based single crystal alloys containing Re.

The hardnesses of the six kinds of Ni–based single crystal alloy samples improved with the increase in the content of Re, which was mainly because Re is a kind of hard metal, the integration of which can enhance the resistance of interface dislocation networks, thus strengthening the resistance of compressive, tensile and shear loads to a certain extent. However, there is no effective means to study how Re improves the hardness of Ni–based single crystal alloys at the micro level; thus, the molecular dynamics method is used to reveal the change mechanism in the following section.

**Figure 7:**
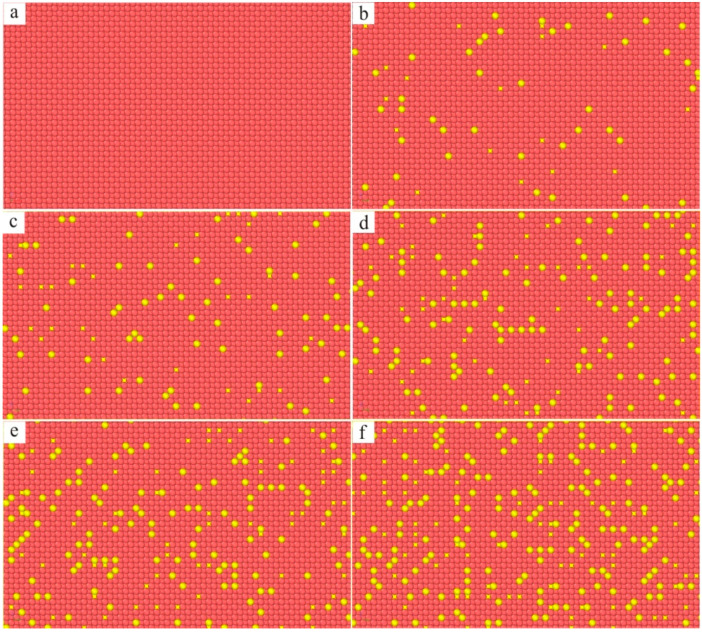
Molecular dynamics models of Ni–based single crystal alloys containing Re: (**a**) 0 wt.% Re; (**b**) 1 wt.% Re; (**c**) 2 wt.% Re; (**d**) 3 wt.% Re; (**e**) 4 wt.% Re; (**f**) 5 wt.% Re.

**Figure 8:**
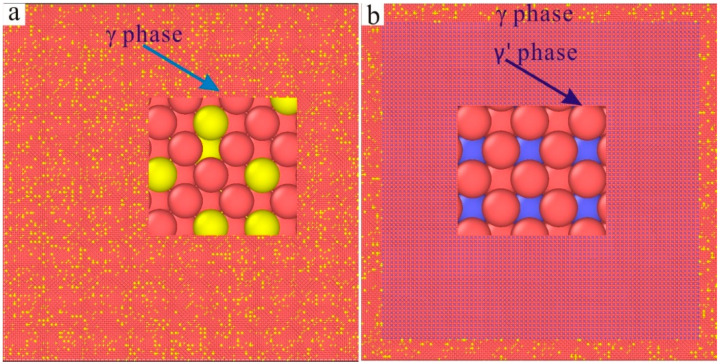
(**a**) γ phase of the molecular dynamics model of Ni–based single crystal alloy containing Re; (**b**) γ′ phase of the molecular dynamics model of Ni–based single crystal alloy containing Re.

**Figure 9:**
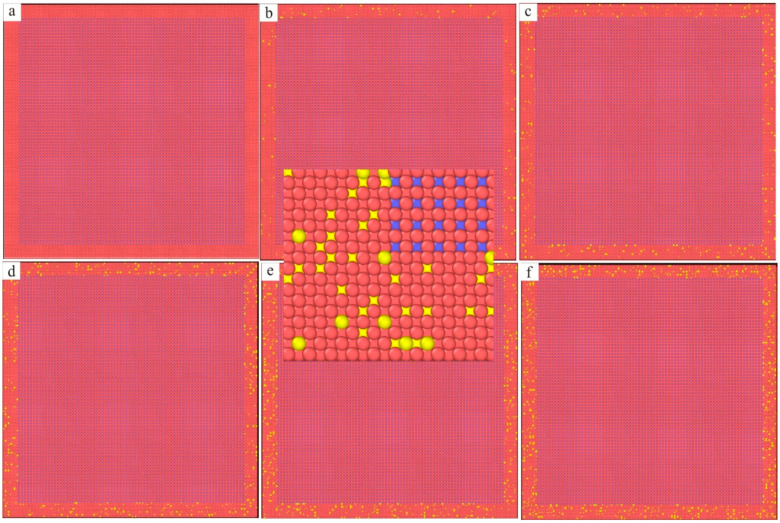
γ/γ′ phase interface of the molecular dynamics models of Ni–based single crystal alloys containing Re: (**a**) 0 wt.% Re; (**b**) 1 wt.% Re; (**c**) 2 wt.% Re; (**d**) 3 wt.% Re; (**e**) 4 wt.% Re; (**f**) 5 wt.% Re.

The three–dimensional molecular dynamics models of Ni–based single crystal alloys containing Re are shown in Figure 7. Figure 7a is a model of an Ni–based single crystal alloy without Re. Figure 7b is the model of an Ni–based single crystal alloy containing 1 wt.% Re, and the yellow atoms in γ phase are Re atoms, which are randomly distributed in the γ phase. Figure 7c–f presents the models of Ni–based single crystal alloys containing 2 wt.% Re, 3 wt.% Re, 4 wt.% Re and 5 wt.% Re, respectively, and the yellow atoms are Re atoms. In the six models, only the atoms of the γ phase can be seen, while the atoms of γ′ phase are inside the model, which can only be seen by cutting the models into sections. The sections of the γ phase and γ′ phase are shown in Figure 8a,b, respectively, and the blue atoms in Figure 8b are Al atoms. Due to the deviation in lattice constants between the γ phase and the γ′ phase, there are mismatched dislocations in the interface of the model, and the dislocation density is an important factor affecting the performance of Ni–based single crystal alloys [10,11,12,13,14,15]. The corresponding γ/γ′ phase interfaces of the six models are shown in Figure 9, and the yellow atoms are Re atoms. There are no Re atoms at the interface in Figure 9a, and Figure 9b–f shows the Re atoms at the interfaces containing 2 wt.% Re, 3 wt.% Re, 4 wt.% Re and 5 wt.% Re, respectively. The potential energy of Re atoms at the phase interface is the lowest, and the potential energy of the surrounding atoms is also reduced by Re atoms, thus enhancing the stability of the system, which can improve the mechanical properties of materials [2].

**Figure 10:**
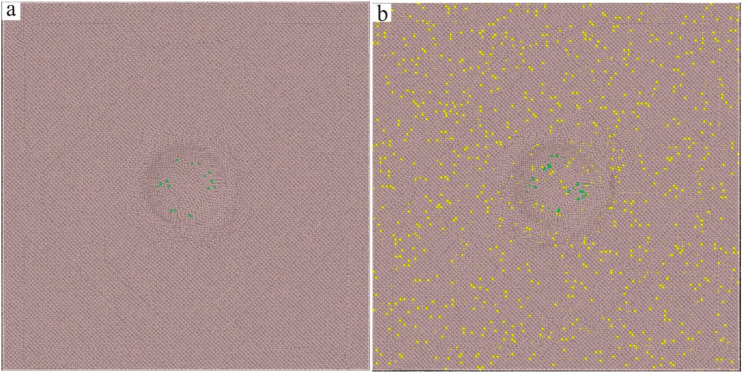
Nano–indentations of the molecular dynamics models of Ni–based single crystal alloys containing Re: (**a**) 0 wt.% Re; (**b**) 1 wt.% Re.

**Figure 11:**
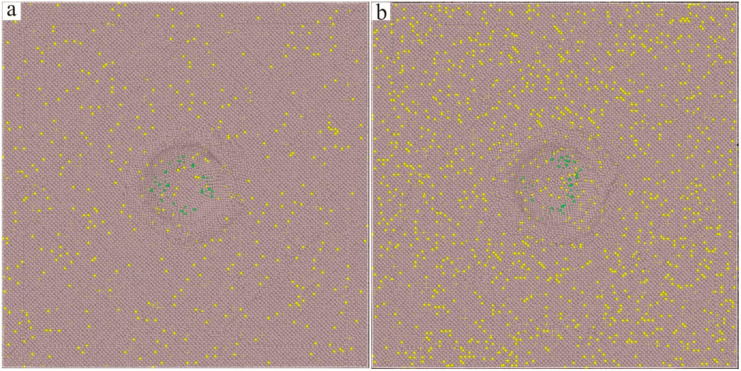
Nano–indentations of the molecular dynamics models of Ni–based single crystal alloys containing Re: (**a**) 2 wt.% Re; (**b**) 3 wt.% Re.

**Figure 12:**
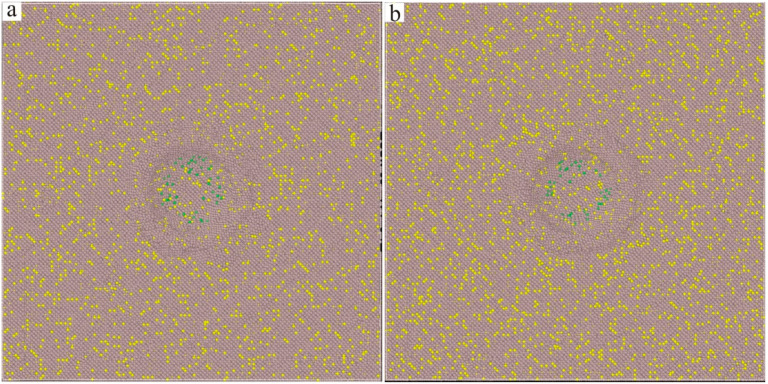
Nano–indentations of the molecular dynamics models of Ni–based single crystal alloys containing Re: (**a**) 4 wt.% Re; (**b**) 5 wt.% Re.

**Figure 13:**
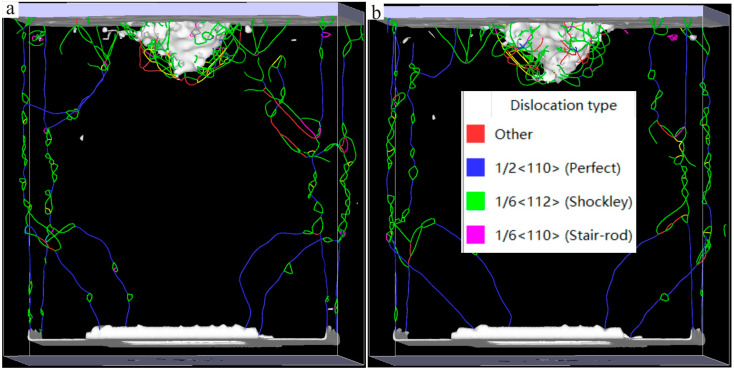
Dislocation distribution states of the molecular dynamics models of Ni–based single crystal alloys containing Re: (**a**) 0 wt.% Re; (**b**) 1 wt.% Re.

**Figure 14:**
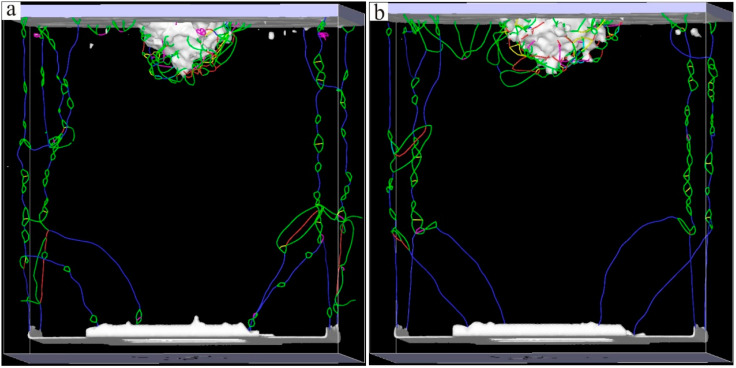
Dislocation distribution states of the molecular dynamics models of Ni–based single crystal alloys containing Re: (**a**) 2 wt.% Re; (**b**) 3 wt.% Re.

**Figure 15:**
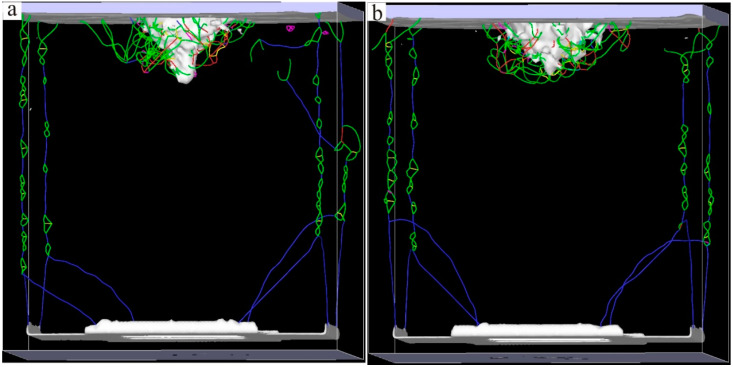
Dislocation distribution states of the molecular dynamics models of Ni–based single crystal alloys containing Re: (**a**) 4 wt.% Re; (**b**) 5 wt.% Re.

The Ni–based single crystal alloy [16,17,18,19] has excellent mechanical properties, which is mainly because of the integration of Re. The hardnesses of the six kinds of Ni–based single crystal alloy samples show certain regularity with the increase in the content of Re. In order to reveal this change mechanism at the micro level, further analysis of the interface dislocation networks was needed. The corresponding nano–indentations of the six models under compressive load are shown in Figure 10, Figure 11 and Figure 12 (The yellow atoms in the model are Re atoms, the green atoms are Al atoms, and the atoms in the matrix are Ni atoms). During the simulation, the model pressure depth was 2.7 nm (the radius of the simulated spherical diamond indenter was 3.5 nm), and the six models exhibited different resistances to the action of compressive loads. Figure 13, Figure 14 and Figure 15 show the dislocation distribution states of the six models at a pressure depth of 2.7 nm, which fully demonstrates the role of the interface dislocation networks. Figure 13a is the dislocation distribution state of the Ni–based single crystal alloy without Re. In this model, the total length of the dislocation loop was 7119 Å. Figure 13b is the dislocation distribution state of the Ni–based single crystal alloy containing 1 wt.% Re, and the total length of the dislocation loop was 6472 Å. The decrease in the total length of the dislocation loop indicates that the integration of Re inhibits the dislocation movement. Figure 14a is the dislocation distribution state of an Ni–based single crystal alloy containing 2 wt.% Re, and the total length of the dislocation loop decreased to 6340 Å, demonstrating that with the increase in the content of Re, the interface dislocation network has a stronger ability to block the dislocation movement. Figure 14b, Figure 15a,b are the dislocation distribution states of Ni–based single crystal alloys containing 3 wt.% Re, 4 wt.% Re and 5 wt.% Re, respectively (the corresponding total lengths of the dislocation loops were 6127 Å, 5954 Å and 5759 Å). The total length of dislocation loop decreased. For the above phenomena, this was mainly because with the increase in the content of Re, the density of the interface dislocation network increased indirectly. However, the dislocations network [20,21] has the blocking ability of γ phase dislocations cutting into the γ′ phase; thus, the ability of the interface dislocation network to suppress the dislocation movement is enhanced. Accordingly, it can be concluded that the increase in the content of Re can strengthen the overall resistance of the model system. 

**Figure 16:**
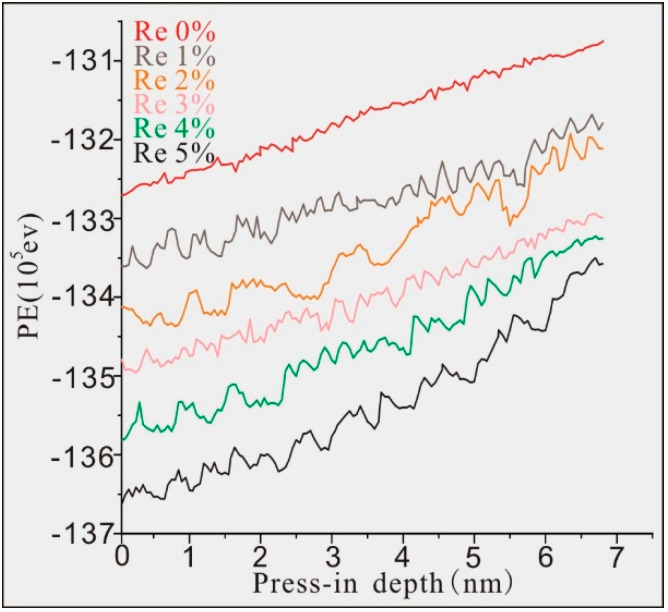
Curves of the potential energy of the model system with pressure depth of the six molecular dynamics models of Ni–based single crystal alloys containing Re.

The atomic mass of Re is 3.17 times that of Ni and 6.90 times that of Al; thus, more energy is needed to push the Re atoms. At the same time, the integration of Re atoms can change the potential energy of the model system, which affects the stability of the model. Figure 16 shows the curves of the potential energy of the model system with the pressure depth of the six molecular dynamics models of Ni–based single crystal alloys containing Re. The initial energies of the six model systems were −13,270,600 ev, −13,371,600 ev, −13,412,400 ev, −13,491,700 ev, −13,581,400 ev and −13,646,300 ev, respectively. It can be found from Figure 16 that the initial potential energy of the model system decreased with the increase in the content of Re, and more elastic potential energies accumulated in the six model system. The main reason for this change trend is that the potential energy of Re atoms at the interface was the lowest, and the potential energy of the surrounding atoms was also reduced by the Re atoms. According to the above analysis, the higher the number of Re atoms, the lower the potential energy of the model system, and thus the better stability of the model.

## 4. Conclusions

To fully reveal the effect mechanism of Re on the interface dislocation networks of Ni–based single crystal alloys, six kinds of Ni–based single crystal alloys with different contents of Re were prepared in this study. The results of the experimental tests and molecular dynamics simulation were as follows:

(1) The mechanical properties of Ni–based single crystal alloys can be enhanced after the integration of Re, and with the increase in the content of Re, the hardnesses of the six kinds of new Ni–based single crystal alloys containing Re showed a nonlinear increasing trend.
(2) The density of the interface dislocation network improved with the increase in the content of Re, and the nonlinear characteristic parameter was proportional to the dislocation density. Thus, the increase in the density of the interface dislocation network was further verified by nonlinear ultrasonic lamb wave tests.
(3) A new model was constructed using the molecular dynamics method, which is closer to the real state of Ni–based single crystal alloys. The number of atoms in this model increased by six to seven times compared with that of the traditional model, and the strengthening mechanism of Re on the square interface dislocation network was analyzed using this model.
(4) After the new Ni–based single crystal alloy was integrated with Re, the interface dislocation network enhanced the blocking ability of γ phase dislocations cutting into the γ′ phase. The analysis of the total lengths of the dislocation loops of the six models showed that the inhibition of the dislocation movement was strengthened by the increase in the number of Re atoms.
(5) The analysis of the atomic potential energy showed that the potential energy of Re atoms at the interface was the lowest, which affected the reduction of the potential energy of other atoms at the interface. Thus, the potential energy of the model system was reduced, and the stability of the model was improved.

## Figures and Tables

**Table 1:** Elemental contents (wt.%) of Ni–based single crystal alloy samples.

Samples	Re	Al	Ti	Cr	Co	Ni	Mo	Ta	W
Sample 1	0	6.16	1.73	9.67	6.10	62.41	2.99	3.70	7.25
Sample 2	1	6.16	1.73	9.67	6.10	61.41	2.99	3.70	7.25
Sample 3	2	6.16	1.73	9.67	6.10	60.41	2.99	3.70	7.25
Sample 4	3	6.16	1.73	9.67	6.10	59.41	2.99	3.70	7.25
Sample 5	4	6.16	1.73	9.67	6.10	58.41	2.99	3.70	7.25
Sample 6	5	6.16	1.73	9.67	6.10	57.41	2.99	3.70	7.25

## Data Availability

The data presented in this study are available upon request from the corresponding author.
